# Recent Progress in Solar-Induced Direct Biomass-to-Electricity Hybrid Fuel Cell Using Microalgae as Feedstocks

**DOI:** 10.3389/fbioe.2021.638971

**Published:** 2021-03-03

**Authors:** Kun-Tao Peng, Xiang Wang, Gong Peng, Lin Yu, Hong-Ye Li

**Affiliations:** ^1^Key Laboratory of Clean Chemistry Technology of Guangdong Regular Higher Education Institutions, School of Chemical Engineering and Light Industry, Guangdong University of Technology, Guangzhou, China; ^2^Key Laboratory of Eutrophication and Red Tide Prevention of Guangdong Higher Education Institutes, College of Life Science and Technology, Jinan University, Guangzhou, China; ^3^School of Civil and Transportation Engineering, Guangdong University of Technology, Guangzhou, China

**Keywords:** microalga, fuel cell, biomass, electricity, solar

## Abstract

Microalgae, as potential biodiesel feedstocks, have been widely reported to accumulate oil via genetic engineering techniques, or environmental stress regulation. Recently, the utilization of fuel cell technology to convert biomass into electricity has attracted much more attention due to its high efficiency, low pollution, low noise by microalgae as feedstocks. Normally, platinum and analogous noble metals as catalysts have been already demonstrated although they still exist lots of shortcomings. This mini review presents an overview of various fuel cell technologies with phosphomolybdic acid as catalysts for sustainable energy by using microalgae. Trends from literatures demonstrate that algal-based fuel cells could efficiently generate electricity, and concurrently produce high value-added products. This critical review can provide guiding suggestions for future study of algal-based energy conversion by fuel cell techniques.

## Introduction

In this resource-exhausted era, the imminent need is to develop sustainable and renewable energy sources since the immoderate fossil fuels extraction and usage which have already caused economic and environmental loss (Li D. W. et al., 2019). Currently, renewable energy sources include solar energy, wind energy, water energy, biomass energy, tidal energy, geothermal energy, and ocean energy, etc. Amongst, biomass energy can be considered as major renewable energy source (Potoènik, 2007). Thus, many efforts focus on the conversion techniques of biomass into electrical energy to reduce the consumption of fossil energy.

Hybrid fuel cell technique, as a typical conversion technology, can generate electrical energy from biomass, however, it still exists several obstacles to be solved. For instance, high working temperature (500–1000°C) should be applied to gasify feedstock (Choudhury et al., 2013; Ruiz et al., 2013). Additionally, energy generation efficiency is relatively low by using fuel cell technique (Ahmad et al., 2013). Furthermore, noble metals which are utilized as catalysts occupy approximately 80% of the total cost (Wee, 2007; Bianchini and Shen, 2009). Recently, polyoxometalates are found to be low-cost catalysts to efficiently generate electrical energy under low temperature by light treatment (Liu et al., 2014). Polyoxometalates also exhibit suitable oxidizing ability which can be recyclable to reduce the catalyst cost (Gaspar et al., 2007). Specifically, the feedstock can be firstly oxidized by polyoxometalates, afterward, polyoxometalates will be recovered by redox reaction with oxygen. In addition, polyoxometalates possess great potential as electron reservoirs because of its typical kegging structure (Mizuno and Misono, 1998). Nowadays, many efforts have been made on the microbial feedstock selection of microbial fuel cell (MFC) system. Microalgae are regarded as an ideal microbial feedstock for MFC, owing to excellent characteristics in biomass production, lipid accumulation, environmental tolerance, etc. (Duffy et al., 2009; Jasny, 2017). Srivastava et al. reported that CaCl_2_ triggered lipid accumulation which reached 45% of dry cell weight in freshwater microalgae (Srivastava and Goud, 2017). The marine diatom *Phaeodactylum tricornutum* exhibits a 2.4-fold increment of neutral lipid under nitrogen-deficiency treatment (Yang et al., 2013). In microalgal-based MFC system, the electricity can be generated by the electrons which is released to the anode during the degradation of microalgae. Furthermore, CO_2_ can be captured by microalgal photosynthesis. Meanwhile, substrates in the anodic compartment can be supplied by microalgae. Therefore, microalgae can be used as a novel type of feedstocks for MFC system.

This mini review not only summarizes the recent study of MCF using microalgae as feedstocks, but also points out the limit factors that affect the power output of fuel cells and the advantages of microalgae-MFC. This review critically provides some promising applications that using microalgae in MFC. One is waste water treatment, that nitrogen and phosphorus in wastewater can be effectively removed by microalgae. The other is reducing the greenhouse effect, that the greenhouse gas carbon dioxide can be captured by microalgae.

## Algal-Based MFC

**FIGURE 1:**
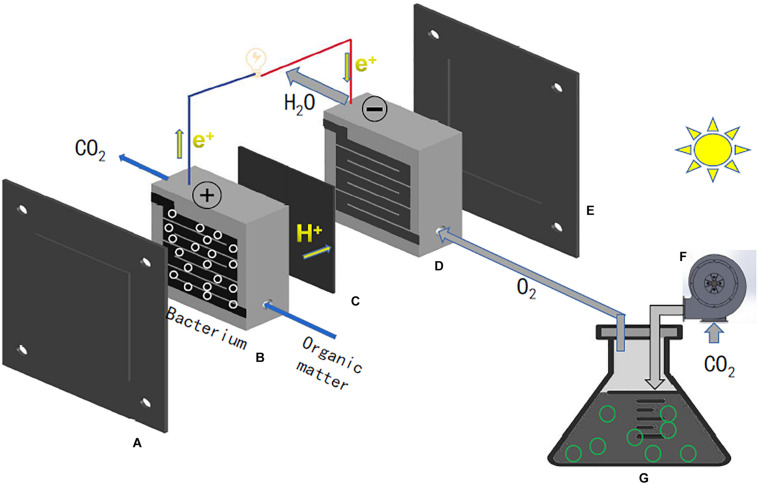
The structure of algal-based MFC. **(A,E)** acrylic plastic end plate, **(B,D)** graphite bipolar plate, **(C)** Nafion polymer exchange membrane, **(F)** pump, and **(G)** microalgae cells.

The basic working principle of MFC is: the organic compound in anode chamber is decomposed by microorganisms to release electrons and protons under anaerobic environment. Previous studies demonstrated that the pivotal step of power generation in MFC is oxygen reduction rate. Several protocols are already updated to enhance the oxygen reduction activities (i.e., stronger reducing salt to catholyte, or continuously pump oxygen to the cathode chamber, apply a catalyst at the cathode). For example, the polyaniline (PANI)-graphene nanosheet (GNS) modified cathode MFC possesses a higher electrical generation capacity than ordinary cathode, due to that PANI has the ability of catalytic oxidation of oxygen at room temperature (Ren et al., 2013). Potassium ferricyanide can effectively improve the power generation of MFC, meanwhile, it can act as an electron acceptor in the cathode chamber to improve oxygen reduction rate (Lay et al., 2015). He et al. (2007) mentioned that the addition of rotating electrodes could improve oxygen utilization in the cathode chamber and result in a higher power production (49 mW/m^2^) compared to a normal cathode system (29 mW/m^2^). In the bacterio-algal microbial system, algae grown in the cathode chamber can continuously release oxygen via photosynthesis under light treatment. Except oxygen reduction rate, electrolytic pH value could also affect the power generation of the MFC. Most MFCs are defined at a neutral pH value which could allow microorganisms to grow efficiently. The real algal-based MFC are shown in Figure 1.

Algal-based MFCs have many advantages, however, they are still required continuous and stable electron donors and acceptors for commercial applications (Mateo et al., 2014). Generally, oxygen serves as an electron acceptor at the cathode, meanwhile the organic compound serves as an electron donor at the anode. In this situation, microalgae in the cathode chamber continuously release oxygen via photosynthesis using carbon dioxide, which can provide a way to solve global greenhouse gas emission (Yabe et al., 2012). In algal-based MFCs, solar energy could easily store as biological energy in primary metabolites and finally convert into electrical energy. Recently, there mainly exists three types of algal-based fuel cells, including single, two and three chambered fuel cells. In single chambered fuel cells, microalgae and other microorganisms are grown in the same chamber which is equipped with an air cathode. In two chambered fuel cells, microalgae and other microorganisms are grown in two different chambers connected with a proton exchange membrane under light source. Similar with two chambered fuel cells, three chambered fuel cells possess an additional middle chamber with salt water, that the power generation was lower than two chambered MFCs (Kokabian and Gude, 2013). Amongst, single chambered fuel cells are the easiest to operate in the laboratory with low-cost. The application of microalgae in MFCs can not only improve its performance, but also have many benefits. In MFCs, microalgae mainly play roles in providing reaction substrates for anode anaerobic microorganisms, and supplying oxygen for cathode reactions through photosynthesis (Baicha et al., 2016). Lobato et al. (2013) has developed a new type of MFCs with microalgae, unlike conventional MFCs system, oxygen no longer have been pumped into the cathode chamber of new system, and cell voltage of new fuel cell has been observed to be the same as conventional MFCs, that means this new system can be performed as well as conventional system. MFC using green microalga *Scenedesmus quadricauda* as a substrate could produce much higher power density than other substrates, owing to dry algae biomass have been used as the substrate in MFC, meanwhile, ultrasonic treatment of *S. quadricauda* is inversely related to power generation capacity (Rashid et al., 2013). Khandelwal et al. (2018) also found that lipid extracted from green microalga *Chlorella vulgaris* as substrate could enhance the power generation of this new MFC at 2.7 W m^–3^ without additional carbon source. Hence, we can infer that the lipids in microalgae are the key to the conversion of biomass energy into electrical energy. *P. tricornutum* with high lipid content and rapid growth can be used as a suitable feedstock for fuel cells. The dissolved oxygen value release was achieved at the maximum value during the 12-h illumination treatment with the similar trend of voltage values (Del Campo et al., 2013). Interestingly, the proton exchange membrane replaced by a biofilm made from *Spirulina platensis* improved strong competitiveness in energy output (Lin et al., 2013). *Cyanobacteria* as a raw material for MFCs could efficiently produce electricity, and remove microcystins to remediate the microcystin-contaminated environments (Yuan et al., 2011). The power output of the MFCs using several typical microalgal species as feedstocks is shown in Table 1.

**TABLE 1:** Electricity production of algal-based MFCs.

**Cathodic contents**	**Anodic contents**	**Power density (mw/m^2^)**	**References**
*Chlorella vulgaris*	Aerobic wastewater sludge	151	Kokabian and Gude, 2013
Mixed algal culture	Municipal wastewater	12.6	Lobato et al., 2013
*Scenedismus obliquus*	Activated sludge	1780	Rashid et al., 2013
*Chlorella vulgaris*	Pre-treated cow manure	67.07	Khandelwal et al., 2018
*Chlorella vulgaris*	Activated sludge	13.5	Del Campo et al., 2013
*Spirulina platensis*	/	10	Lin et al., 2013
Blue green algae	Anaerobic wastewater sludge	114	Yuan et al., 2011

### The Advantage of Algal-Based MFCs

Algal-based MFCs have plenty of advantages, such as high electricity generation, strong wastewater treatment ability, high valued bioproducts, carbon dioxide assimilation, and oxygen production (Saba et al., 2017). The concentrations of nitrogen and phosphorus in wastewater are extremely high, which often cause water eutrophication. Many studies mentioned that algal-based fuel cells could be used for wastewater treatment since wastewater contains large amounts of degradable organic compounds (Zhang et al., 2011; Commault et al., 2017). Wastewater contains monosaccharides, sugar derivatives, polyalcohols, amino acids, organic acids, alcohols, and nitrogenous heterocyclic compounds, etc., which could serve as substrates to provide electrons. Compared to conventional wastewater treatments, algal-based fuel cell system could save lots of energy and cost. The chemical oxygen demand (COD) reduction of water is used as an important criterion for testing the effect of wastewater treatment. As expected, over 70% COD of wastewater could be eliminated in MFC system (Gadhamshetty et al., 2013; Cui et al., 2014; Mohan et al., 2014). An algae biofilm microbial fuel cell (ABMFC) was invented by Yang et al., and its performance was much better than MFC or AB alone. The COD of ABMFC reached 80.2%, and the power generation was 18% higher than MFC (Yang et al., 2018). In addition, the removal rates of nitrogen and phosphorus in wastewater was 96.0, 91.5%, respectively.

Constructed wetlands have received widespread attention in recent years as a green and low-cost way to treat wastewater (Vymazal, 2011). Srivastava et al. (2020a, b), combined technology of MFC technique and constructed wetland for improving wastewater treatment performance (Srivastava and Goud, 2017). Compared with using MFCs alone to treat wastewater, algal-based MFCs could use nutrients in wastewater as their nitrogen and phosphorus sources to produce biomass, thereby effectively removing nitrogen and phosphorus in wastewater.

Algal-based MFCs could also produce some high value-added bioactive compounds while generating electricity, such as polyunsaturated fatty acid (PUFA), carotenoids including β-carotene, lutein, etc. Carotenoids have been widely used in antioxidants, pharmaceuticals, and nutraceuticals, as they have important healthy properties to human, e.g., they can promote the release of anti-tumor factors by cells (Maiani et al., 2009). Carotenoids content can be increased significantly under the light treatment in photosynthetic algae MFC system (Gouveia et al., 2014).

### The Limit Factors Affecting the Fuel Cell Power Output

#### Fuel Cell Catalyst

As an important factor affecting the fuel cell power output, fuel cell catalysts have been increasingly researched in recent years. Liu et al. (2016) employed polyoxometallates such as phosphomolybdic acid as catalysts to electrolyze biomass for hydrogen production consume which is only 16.7% of the energy consumed for the reported water electrolysis. Hydrogen is the cleanest fuel in the world, and has the highest energy density (143 KJ Kg^–1^), which the energy produced by burning 1 kg of hydrogen is equivalent to the energy produced by burning 2.63 kg of gasoline (Hoffert et al., 2002; Turner, 2004; Parthasarathy and Narayanan, 2014). Five common methods, such as thermo-conversion, photo-electrochemical conversion, fermentation, and electrolysis which can convert biomass into hydrogen (Turner et al., 2008). Compared with traditional methods, Deng et al. reported a method which can generate hydrogen more efficiently and solve some shortcomings. The high efficiency hydrogen evolution from native biomass electrolysis are calculated using the following equations:

(1) $$\mathrm{Biomass}+H_{2}\,O+{\mathrm{POM}}_{\left(\text{OX}\right)}\,\overset{\Delta \,\mathrm{or}\,\mathrm{hv}}{\to }\mathrm{Degradation}\,\mathrm{products}+\,{\text{CO}}_{2}+\text{H}-{\text{POM}}_{\left(\text{Red}\right)}$$

(2) $${\text{H-POM}}_{\left(\text{Red}\right)}\,\overset{\text{Anode}}{\to }{\text{POM}}_{\left(\text{OX}\right)}+{\text{H}}^{+}+{\text{e}}^{-}$$

(3) $${\text{H}}^{+}+{\text{e}}^{-}\,\overset{\text{Cathode}}{\to }1/2\,{\text{H}}_{2}$$

The traditional treatment of wheat straw is *in situ* burning, which not only causes a lot of energy loss, but also pollutes the air. Previous reports showed that phosphomolybdic acid and ferric ions as high-efficiency electron carriers and oxidants can assist wheat straw to produce bioethanol and electricity (Ding et al., 2017). This method is a good solution to the problems of air pollution and energy waste. Biomass pretreatment is a key step in the degradation of lignocellulose to produce ethanol, etc. Compared with traditional biomass pretreatment, such as alkaline saponification, oxidative delignification, solvent extraction, and sulfite pulping, pretreatment of wheat straw by phosphomolybdic acid (PMo_12_) can effectively improve cellulose hydrolyzability and ethanol production (Zhao et al., 2009; Zhu et al., 2009; Singh et al., 2014; Kim et al., 2016). A polyoxometalate coupled graphene oxide-Nafion composite membrane for fuel cells was developed to show a 4-fold higher maximum fuel cell power density (Kim et al., 2015). When FeCl_3_ was introduced into the Liquid catalytic fuel cell as co-catalyst, it could replace 80% of the raw catalyst (polyoxometalates), and the performance of the fuels could keep the same level (Xu et al., 2017). Hence, the addition of Fe^3+^ can promote the catalytic ability of phosphomolybdic acid, as the addition of Fe^3+^ promotes the transfer of electrons to oxygen. Therefore, we can use phosphomolybdic acid and other polyoxometalates as fuel cell catalysts to promote the conversion of chemical energy, solar energy, biomass energy into electrical energy.

### Light and CO_2_ Influence on Power Production

It is considered that the oxygen release by microalgae influenced by light and CO_2_ concentration in the anode chamber is the main factor to affect the production of electrical energy. The electricity generation of bacterio-algal MFCs under 6–12 W light intensity power exhibits higher than that under 18–26 W. Furthermore, the anodic chambers have been covered which shows higher voltage, power density, Coulombic efficiency and specific power than the uncovered anodic chambers (Juang et al., 2012). Gouveia et al. (2014) studied the effect of light intensity on photosynthetic microalgal MFC, the power generation increased by 6-folds when the light intensity increased from 26 to 96 μE/(m^2^s), and the production of carotenoids in the cathode compartment also increased. It is well known that light is an important factor affecting photosynthesis, so, finding a suitable light intensity and light time is the key to the commercialization of microalgal MFC. In addition, Bazdar et al. (2018) found the light intensity between 5000 and 6500 lx is the optimal light range for *Chlorella vulgaris* photosynthesis and maximum energy production in the photosynthetic microalgae MFC. Furthermore, this study showed that increasing the light time has a positive effect on the output of electric energy, the maximum power density in the light/dark regimes of 24/00 h was 12.7 and 74.8% higher than 16/8 and 12/12 h, respectively. CO_2_ concentration is another important factor to promote the algal photosynthesis. Currently, microalgae in the cathode chamber utilize CO_2_ degraded from the organic substrate to ensure the effective operation of microalgae microbial fuel cell system (Wang et al., 2010; Del Campo et al., 2013). Li M. et al. (2019) developed a new algal-based MFC which 10% CO_2_ was pumped into cathode chamber, and the performance of this new fuel cell was better than photosynthetic algae MFC and bubbling photobioreactor in bioenergy production and lipid production. Moreover, the continuous pumping of CO_2_ can not only be used as a carbon source for algae growth, but also adjust the pH of the catholyte. Compared with other nitrogen sources, the new fuel cell uses urea as nitrogen source to better capture CO_2_, generate electricity, and produce high value-added biological products.

## Conclusion and Future Perspectives

Algal-based fuel cell has been extensively developed in laboratory scale with good performance, however, it still cannot be utilized in industrial applications due to several limited factors. The first reason is that compared with traditional fossil fuels, microalgae biodiesel is more expensive, which is the most important factor limiting the commercialization of biodiesel. Microalgal oil is almost three to four times more expensive than plant oil (Rastogi et al., 2018). Compared with traditional plant oils, the cost of nutrients and substrates required for the growth of microalgae is high. In addition, the production of biodiesel and biological products using algae as raw materials requires pretreatment of algae, which greatly increases the cost (Hoh et al., 2016). Furthermore, the fuel cell generally requires a nafion proton exchange membrane, however, it is too expensive, about $1500/m^2^ to $3000/m^2^, and such high cost limits the feasibility in scaled-up systems. Khandelwal et al. (2020) developed an outdoor alga assisted MFC, which used low-cost materials including rock phosphate blended clayware and low-density polyethylene bags to replace the nafion proton exchange membrane, and the cost of this fuel cell was only $11.225. The second barrier is how to effectively balance the growth of algae and energy production, and find an optimal solution, for example, the carbon source, nitrogen source, and light intensity which are required for algae growth. The final barrier is the problem of purification of a large amount of value-added biological products produced by MFC has not been solved. Therefore, in order to solve the above obstacles, a new type of MFCs are needed further innovation, with a view to achieve commercialization in the future. For example, genetically engineered microalgae by gene knockout, gene overexpression or other genetic methods to enhance the overall productivity of microalgae for MFCs.

In the future, the microalgae will be used as the feedstocks of fuel cells, and the phosphomolybdic acid (POM) used as the catalyst, and the structure as shown in Figure 1. Through this new type of microalgae fuel cell, we can get electricity efficiently in normal or even low temperature environments. Some harmful red tide microalgae are used as the feedstocks, such as *Prorocentrium lima* and *Alexandrium tamarense*. This not only provides a method for controlling red tide, but also produces electricity.

## Author Contributions

K-TP, LY, and H-YL conceived of and designed experiments. XW and GP contributed to literature analysis. K-TP, LY, and H-YL wrote the manuscript. All authors contributed to the article and approved the submitted version.

## Conflict of Interest

The authors declare that the research was conducted in the absence of any commercial or financial relationships that could be construed as a potential conflict of interest.

## References

[B1] Ahmad F. Atiyeh M. N. Stephanopoulos GN. Pereira B. (2013). A review of cellulosic microbial fuel cells: performance and challenges. *Biomass Bioenergy* 56 179–188. 10.1016/j.biombioe.2013.04.006

[B2] Baicha Z. Salar-García M. J. Ortiz-Martínez V. M. Hernández-Fernández F. J. de los Ríos A. P. Labjar N. (2016). A critical review on microalgae as an alternative source for bioenergy production: a promising low cost substrate for microbial fuel cells. *Fuel Process. Technol.* 154 104–116. 10.1016/j.fuproc.2016.08.017

[B3] Bazdar E. Roshandel R. Yaghmaei S. Mardanpour M. M. (2018). The effect of different light intensities and light/dark regimes on the performance of photosynthetic microalgae microbial fuel cell. *Bioresour. Technol.* 261 350–360. 10.1016/j.biortech.2018.04.026 29679853

[B4] Bianchini C. Shen P. K. (2009). Palladium-based electrocatalysts for alcohol oxidation in half cells and in direct alcohol fuel cells. *Chem. Rev.* 109 4183–4206. 10.1021/cr9000995 19606907

[B5] Choudhury A. Chandra H. Arora A. (2013). Application of solid oxide fuel cell technology for power generation—a review. *Renew. Sustain. Energy Rev.* 20 430–442.

[B6] Commault A. S. Laczka O. Siboni N. Tamburic B. Crosswell J. R. Seymour J. R. (2017). Electricity and biomass production in a bacteria-Chlorella based microbial fuel cell treating wastewater. *J. Power Sources* 356 299–309. 10.1016/j.jpowsour.2017.03.097

[B7] Cui Y. Rashid N. Hu N. Rehman M. S. U. Han J.-I. (2014). Electricity generation and microalgae cultivation in microbial fuel cell using microalgae-enriched anode and bio-cathode. *Energy Conv. Manag.* 79 674–680. 10.1016/j.enconman.2013.12.032

[B8] Del Campo A. G. Cañizares P. Rodrigo M. A. Fernández F. J. Lobato J. (2013). Microbial fuel cell with an algae-assisted cathode: a preliminary assessment. *J. Power Sources* 242 638–645. 10.1016/j.jpowsour.2013.05.110

[B9] Ding Y. Du B. Zhao X. Zhu J. Y. Liu D. (2017). Phosphomolybdic acid and ferric iron as efficient electron mediators for coupling biomass pretreatment to produce bioethanol and electricity generation from wheat straw. *Bioresour. Technol.* 228 279–289. 10.1016/j.biortech.2016.12.109 28081526

[B10] Duffy J. E. Canuel E. A. Adey W. Swaddle J. P. (2009). Biofuels: algae. *Science* 326 1345–1345. 10.1126/science.326.5958.1345-a 19965739

[B11] Gadhamshetty V. Belanger D. Gardiner C.-J. Cummings A. Hynes A. (2013). Evaluation of Laminaria-based microbial fuel cells (LbMs) for electricity production. *Bioresour. Technol.* 127 378–385. 10.1016/j.biortech.2012.09.079 23138060

[B12] Gaspar A. R. Gamelas J. A. F. Evtuguin D. V. Neto C. P. (2007). Alternatives for lignocellulosic pulp delignification using polyoxometalates and oxygen: a review. *Chem. Inform.* 38 717–730. 10.1039/b607824a

[B13] Gouveia L. Neves C. Sebastião D. Nobre B. P. Matos C. T. (2014). Effect of light on the production of bioelectricity and added-value microalgae biomass in a photosynthetic alga microbial fuel cell. *Bioresour. Technol.* 154 171–177. 10.1016/j.biortech.2013.12.049 24388957

[B14] He Z. Shao H. Angenent L. T. (2007). Increased power production from a sediment microbial fuel cell with a rotating cathode. *Biosens. Bioelectron.* 22 3252–3255. 10.1016/j.bios.2007.01.010 17314039

[B15] Hoffert M. I. Caldeira K. Benford G. Criswell D. R. Green C. Herzog H. (2002). Advanced technology paths to global climate stability: energy for a greenhouse planet. *science* 298 981–987. 10.1126/science.1072357 12411695

[B16] Hoh D. Watson S. Kan E. (2016). Algal biofilm reactors for integrated wastewater treatment and biofuel production: a review. *Chem. Eng. J.* 287 466–473. 10.1016/j.cej.2015.11.062

[B17] Jasny B. R. (2017). Bulking up algae for biofuels. *Science* 357 160–161. 10.1126/science.357.6347.160-e 28706061

[B18] Juang D. Lee C. Hsueh S. (2012). Comparison of electrogenic capabilities of microbial fuel cell with different light power on algae grown cathode. *Bioresour. Technol.* 123 23–29. 10.1016/j.biortech.2012.07.041 22929741

[B19] Khandelwal A. Chhabra M. Yadav P. (2020). Performance evaluation of algae assisted microbial fuel cell under outdoor conditions. *Bioresour. Technol.* 310 123418. 10.1016/j.biortech.2020.123418 32353768

[B20] Khandelwal A. Vijay A. Dixit A. Chhabra M. (2018). Microbial fuel cell powered by lipid extracted algae: a promising system for algal lipids and power generation. *Bioresour. Technol.* 247 520–527. 10.1016/j.biortech.2017.09.119 28972905

[B21] Kim J. S. Lee Y. Kim T. H. (2016). A review on alkaline pretreatment technology for bioconversion of lignocellulosic biomass. *Bioresour. Technol.* 199 42–48. 10.1016/j.biortech.2015.08.085 26341010

[B22] Kim Y. Ketpang K. Jaritphun S. Park J. S. Shanmugam S. (2015). A polyoxometalate coupled graphene oxide–Nafion composite membrane for fuel cells operating at low relative humidity. *J. Mater. Chem. A* 3 8148–8155. 10.1039/c5ta00182j

[B23] Kokabian B. Gude V. G. (2013). Photosynthetic microbial desalination cells (PMDCs) for clean energy, water and biomass production. *Environ. Sci. Process. Impacts* 15 2178–2185. 10.1039/c3em00415e 24154718

[B24] Lay C.-H. Kokko M. E. Puhakka J. A. (2015). Power generation in fed-batch and continuous up-flow microbial fuel cell from synthetic wastewater. *Energy* 91 235–241. 10.1016/j.energy.2015.08.029

[B25] Li D. W. Balamurugan S. Yang Y. F. Zheng J. W. Huang D. Zou L. G. (2019). Transcriptional regulation of microalgae for concurrent lipid overproduction and secretion. *Sci. Adv.* 5:eaau3795. 10.1126/sciadv.aau3795 30729156 PMC6353619

[B26] Li M. Zhou M. Luo J. Tan C. Tian X. Su P. (2019). Carbon dioxide sequestration accompanied by bioenergy generation using a bubbling-type photosynthetic algae microbial fuel cell. *Bioresour. Technol.* 280 95–103. 10.1016/j.biortech.2019.02.038 30763866

[B27] Lin C.-C. Wei C.-H. Chen C.-I. Shieh C.-J. Liu Y.-C. (2013). Characteristics of the photosynthesis microbial fuel cell with a *Spirulina platensis* biofilm. *Bioresour. Technol.* 135 640–643. 10.1016/j.biortech.2012.09.138 23186678

[B28] Liu W. Cui Y. Du X. Zhang Z. Chao Z. Deng Y. (2016). High efficiency hydrogen evolution from native biomass electrolysis. *Energy Environ. Sci.* 9 467–472. 10.1039/c5ee03019f

[B29] Liu W. Mu W. Liu M. Zhang X. Cai H. Deng Y. (2014). Solar-induced direct biomass-to-electricity hybrid fuel cell using polyoxometalates as photocatalyst and charge carrier. *Nat. Commun.* 5 1–8. 10.1038/ncomms4208 24504242

[B30] Lobato J. del Campo A. G. Fernández F. J. Cañizares P. Rodrigo M. A. (2013). Lagooning microbial fuel cells: a first approach by coupling electricity-producing microorganisms and algae. *Appl. Energy* 110 220–226. 10.1016/j.apenergy.2013.04.010

[B31] Maiani G. Periago Castón M. J. Catasta G. Toti E. Cambrodón I. G. Bysted A. (2009). Carotenoids: actual knowledge on food sources, intakes, stability and bioavailability and their protective role in humans. *Mol. Nutr. Food Res.* 53 S194–S218. 19035552 10.1002/mnfr.200800053

[B32] Mateo S. Del Campo A. G. Cañizares P. Lobato J. Rodrigo M. Fernandez F. (2014). Bioelectricity generation in a self-sustainable microbial solar cell. *Bioresour. Technol.* 159 451–454. 10.1016/j.biortech.2014.03.059 24709531

[B33] Mizuno N. Misono M. (1998). Heterogeneous catalysis. *Chem. Rev.* 98 199–218. 11851503 10.1021/cr960401q

[B34] Mohan S. V. Srikanth S. Chiranjeevi P. Arora S. Chandra R. (2014). Algal biocathode for in situ terminal electron acceptor (TEA) production: synergetic association of bacteria–microalgae metabolism for the functioning of biofuel cell. *Bioresour. Technol.* 166 566–574. 10.1016/j.biortech.2014.05.081 24953968

[B35] Parthasarathy P. Narayanan K. S. (2014). Hydrogen production from steam gasification of biomass: influence of process parameters on hydrogen yield–a review. *Renew. Energy* 66 570–579. 10.1016/j.renene.2013.12.025

[B36] Potoènik J. (2007). Renewable energy sources and the realities of setting an energy agenda. *Science* 315 810–811. 10.1126/science.1139086 17289990

[B37] Rashid N. Cui Y.-F. Rehman M. S. U. Han J.-I. (2013). Enhanced electricity generation by using algae biomass and activated sludge in microbial fuel cell. *Sci. Total Environ.* 456 91–94. 10.1016/j.scitotenv.2013.03.067 23584037

[B38] Rastogi R. P. Pandey A. Larroche C. Madamwar D. (2018). Algal green energy–R&D and technological perspectives for biodiesel production. *Renew. Sustain. Energy Rev.* 82 2946–2969.

[B39] Ren Y. Pan D. Li X. Fu F. Zhao Y. Wang X. (2013). Effect of polyaniline−graphene nanosheets modified cathode on the performance of sediment microbial fuel cell. *J. Chem. Technol. Biotechnol.* 88 1946–1950. 10.1002/jctb.4146

[B40] Ruiz J. A. Juárez M. Morales M. Mendívil M. Muñoz P. (2013). Biomass gasification for electricity generation: review of current technology barriers. *Renew. Sustain. Energy Rev.* 18 174–183. 10.1016/j.rser.2012.10.021

[B41] Saba B. Christy A. D. Yu Z. Co A. C. (2017). Sustainable power generation from bacterio-algal microbial fuel cells (MFCs): an overview. *Renew. Sustain. Energy Rev.* 73 75–84. 10.1016/j.rser.2017.01.115

[B42] Singh R. Shukla A. Tiwari S. Srivastava M. (2014). A review on delignification of lignocellulosic biomass for enhancement of ethanol production potential. *Renew. Sustain. Energy Rev.* 32 713–728. 10.1016/j.rser.2014.01.051

[B43] Srivastava G. Goud V. V. (2017). Salinity induced lipid production in microalgae and cluster analysis (ICCB 16-BR_047). *Bioresour. Technol.* 242 244–252. 10.1016/j.biortech.2017.03.175 28390788

[B44] Srivastava P. Abbassi R. Garaniya V. Lewis T. Yadav A. K. (2020a). Performance of pilot-scale horizontal subsurface flow constructed wetland coupled with a microbial fuel cell for treating wastewater. *J. Water Process Eng.* 33:100994. 10.1016/j.jwpe.2019.100994

[B45] Srivastava P. Yadav A. K. Garaniya V. Lewis T. Abbassi R. Khan S. J. (2020b). Electrode dependent anaerobic ammonium oxidation in microbial fuel cell integrated hybrid constructed wetlands: a new process. *Sci. Total Environ.* 698:134248. 10.1016/j.scitotenv.2019.134248 31494423

[B46] Turner J. Sverdrup G. Mann M. K. Maness P. C. Kroposki B. Ghirardi M. (2008). Renewable hydrogen production. *Int. J. Energy Res.* 32 379–407.

[B47] Turner J. A. (2004). Sustainable hydrogen production. *Science* 305 972–974. 15310892 10.1126/science.1103197

[B48] Vymazal J. (2011). Constructed wetlands for wastewater treatment: five decades of experience. *Environ. Sci. Technol.* 45 61–69. 10.1021/es101403q 20795704

[B49] Wang X. Feng Y. Liu J. Lee H. Li C. Li N. (2010). Sequestration of CO2 discharged from anode by algal cathode in microbial carbon capture cells (MCCs). *Biosens. Bioelectron.* 25 2639–2643. 10.1016/j.bios.2010.04.036 20547055

[B50] Wee J.-H. (2007). Applications of proton exchange membrane fuel cell systems. *Renew. Sustain. Energy Rev.* 11 1720–1738.

[B51] Xu F. Li H. Liu Y. Jing Q. (2017). Advanced redox flow fuel cell using ferric chloride as main catalyst for complete conversion from carbohydrates to electricity. *Sci. Rep.* 7 1–9. 28698567 10.1038/s41598-017-05535-2 PMC5505984

[B52] Yabe K. Shinoda Y. Seki T. Tanaka H. Akisawa A. (2012). Market penetration speed and effects on CO2 reduction of electric vehicles and plug-in hybrid electric vehicles in Japan. *Energy Policy* 45 529–540. 10.1016/j.enpol.2012.02.068

[B53] Yang Z. Pei H. Hou Q. Jiang L. Zhang L. Nie C. (2018). Algal biofilm-assisted microbial fuel cell to enhance domestic wastewater treatment: nutrient, organics removal and bioenergy production. *Chem. Eng. J.* 332 277–285. 10.1016/j.cej.2017.09.096

[B54] Yang Z.-K. Niu Y.-F. Ma Y.-H. Xue J. Zhang M.-H. Yang W.-D. (2013). Molecular and cellular mechanisms of neutral lipid accumulation in diatom following nitrogen deprivation. *Biotechnol. Biofuels* 6:67. 10.1186/1754-6834-6-67 23642220 PMC3662598

[B55] Yuan Y. Chen Q. Zhou S. Zhuang L. Hu P. (2011). Bioelectricity generation and microcystins removal in a blue-green algae powered microbial fuel cell. *J. Hazard. Mater.* 187 591–595. 10.1016/j.jhazmat.2011.01.042 21295401

[B56] Zhang Y. Noori J. S. Angelidaki I. (2011). Simultaneous organic carbon, nutrients removal and energy production in a photomicrobial fuel cell (PFC). *Energy Environ. Sci.* 4 4340–4346. 10.1039/c1ee02089g

[B57] Zhao X. Cheng K. Liu D. (2009). Organosolv pretreatment of lignocellulosic biomass for enzymatic hydrolysis. *Appl. Microbiol. Biotechnol.* 82:815. 10.1007/s00253-009-1883-1 19214499

[B58] Zhu J. Pan X. Wang G. Gleisner R. (2009). Sulfite pretreatment (SPORL) for robust enzymatic saccharification of spruce and red pine. *Bioresour. Technol.* 100 2411–2418. 10.1016/j.biortech.2008.10.057 19119005

